# Queer- and trans-inclusive faculty hiring—A call for change

**DOI:** 10.1371/journal.pbio.3002919

**Published:** 2024-11-22

**Authors:** J. L. Weissman, Callie R. Chappell, Bruno Francesco Rodrigues de Oliveira, Natalya Evans, Anna C. Fagre, Desiree Forsythe, Steven A. Frese, Rachel Gregor, Suzanne L. Ishaq, Juliet Johnston, Bittu K. R., Shayle B. Matsuda, Sam McCarren, Melanie Ortiz Alvarez de la Campa, Troy A. Roepke, Nasa. Sinnott-Armstrong, Cora S. Stobie, Lauren Talluto, José M. Vargas-Muñiz, The Advancing Queer and Trans Equity in Science (AQTES) Consortium

**Affiliations:** 1 Department of Ecology & Evolution, Stony Brook University, Stony Brook, New York State, United States of America; 2 Institute for Advanced Computational Science, Stony Brook University, Stony Brook, New York State, United States of America; 3 Department of Biology, The City College of New York, New York, New York State, United States of America; 4 Department of Biology, Stanford University, Stanford, California, United States of America; 5 Department of Microbiology and Parasitology, Biomedical Institute, Fluminense Federal University, Niterói, Rio de Janeiro, Brazil; 6 Marine Science Institute, University of California, Santa Barbara, California, United States of America; 7 Center for Vector-Borne Infectious Diseases, Colorado State University, Fort Collins, Colorado, United States of America; 8 Department of Biology, Santa Clara University, Santa Clara, California, United States of America; 9 Department of Nutrition, University of Nevada, Reno, Nevada, United States of America; 10 University of Nevada, Reno School of Medicine, Reno, Nevada, United States of America; 11 Department of Civil and Environmental Engineering, Massachusetts Institute of Technology, Cambridge, Massachusetts, United States of America; 12 School of Food and Agriculture, University of Maine, Orono, Maine, United States of America; 13 Natural Science, Hampshire College, Amherst, Massachusetts, United States of America; 14 Department of Biology, Trivedi School of Biosciences, Ashoka University, Sonipat, Haryana, India; 15 Department of Psychology, Ashoka University, Sonipat, Haryana, India; 16 Conservation Research Department, John G. Shedd Aquarium, Chicago, Illinois, United States of America; 17 Department of Molecular and Cell Biology, University of Cape Town, Cape Town, South Africa; 18 Department of Botany and Zoology, Stellenbosch University, Stellenbosch, South Africa; 19 Department of Molecular Microbiology and Immunology, Brown University, Providence, Rhode Island, United States of America; 20 Department of Animal Sciences, School of Environmental and Biological Sciences, Rutgers University, New Brunswick, New Jersey, United States of America; 21 Herbold Computational Biology Program, Public Health Sciences Division, Fred Hutchinson Cancer Center, Seattle, Washington, United States of America; 22 Division of Herpetology, Department of Animal and Plant Systematics, National Museum Bloemfontein, Bloemfontein, South Africa; 23 Department of Zoology, University of the Free State, Bloemfontein, South Africa; 24 Department of Ecology, University of Innsbruck, Innsbruck, Austria; 25 Department of Biological Sciences, Virginia Polytechnic Institute and State University, Blacksburg, Virginia, United States of America

## Abstract

Queer and trans scientists face varied and systemic barriers to their professional success, resulting in a relative absence from faculty ranks at many institutions. This Perspective calls for a change in faculty hiring practices and present concrete guidance to make it a more inclusive process.

## Teaser

As queer and trans scientists, we face varied and systemic barriers to our professional success, resulting in our relative absence from faculty ranks at many institutions. In this Perspective, we call for a change in faculty hiring practices and present concrete guidance to make it a more inclusive process.

Queer and transgender (trans) scientists face documented systemic challenges across the sciences. We are more likely to experience harassment, burnout, social exclusion, unsupportive working environments, the absence of role models, and biased stereotypes [1–3]. At the same time, we work against a global political and legal backdrop where anti-queer and anti-trans legislation is being passed at a record rate [4,5]. Unsurprisingly, queer and trans trainees have a higher attrition rate from the sciences than our peers.

As a result, relatively few queer and trans scientists have passed through the gauntlet of the faculty job search to become faculty, a step that is key to our long-term persistence in academia. Our lack of representation creates a self-reinforcing cycle wherein early-career queer and trans scientists do not see our needs considered in established processes and power structures. This lack of institutional power disproportionately impacts those of us who have multiple intersecting marginalized identities. Yet, we do not accept this status quo quietly. Early-career scientists have called for the establishment of professional support and advocacy networks for queer and trans researchers, as well as the implementation of institutional policies to protect us [6–9].

We urge departments and institutions to take these demands seriously and to take concrete steps to support queer and trans scientists at all levels. Too often, we have seen a lack of expertise in issues facing queer and trans scientists used as an excuse for administrative inaction. As a way forward, we have developed comprehensive guidance for institutions hoping to recruit queer and trans faculty members, with the ultimate goal of ensuring that queer and trans voices are given a platform in the academy [10].

We are Advancing Queer and Trans Equity in Science (AQTES), an international working group of nearly 50 queer and trans biologists and environmental scientists, currently based in at least 12 different countries on 5 continents, who have expertise and deep personal experience in this topic. Many of us are early-career and are currently on, or have recently been on, the faculty job market. Our recommendations to search committees on the development of inclusive and equitable faculty selection policies are based on our personal experiences on the job market and our shared expertise in building queer and trans inclusive spaces and processes [6,7,10].

Our guidelines for running a queer- and trans-inclusive faculty hiring process were developed through an iterative process of collaborative community engagement. Successive drafts were posted publicly on social media platforms and distributed through personal and professional networks and academic society listservs alongside a call for feedback and an opportunity for community members to join our team. Through this process, our set of named contributors quadrupled in size and a diversity of voices brought nuance to a variety of complex issues facing queer and trans scientists around the world. We have chosen to publish our guidelines as a whitepaper [10], a format that allows the work to exist as a living document, with version-controlled revisions to be posted after planned quinquennial (5 year) public comment and review to ensure that our guidance tracks a constantly changing scientific and social landscape. We provide a concise overview below and encourage academics and hiring committees to read the full whitepaper (doi.org/10.32942/X2J310) [10].

**Fig 1:**
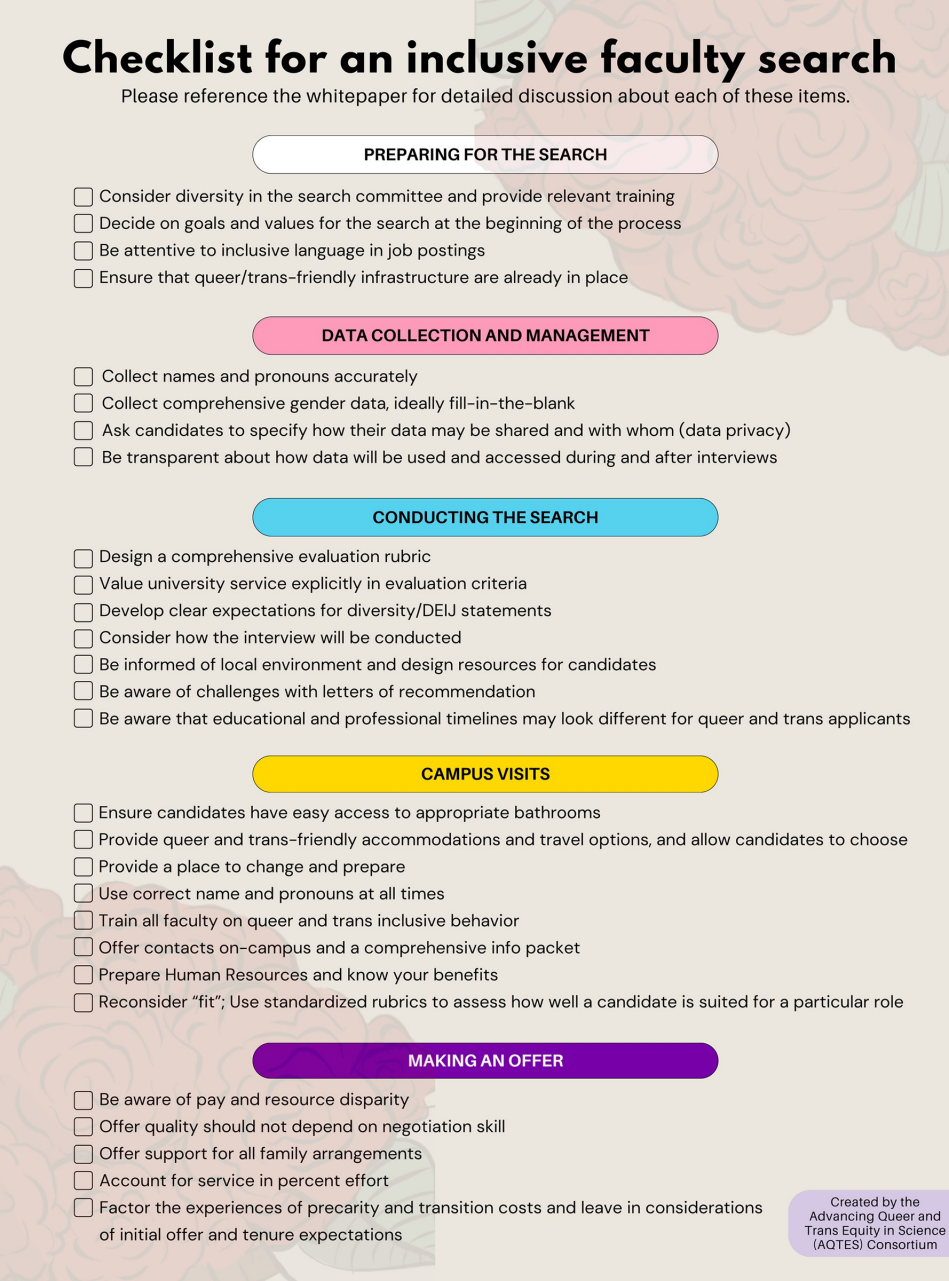
Checklist for running a queer- and trans-inclusive faculty search. Please reference the whitepaper [10] for detailed discussions about each of these items.

Running an inclusive faculty search begins long before a job is posted and includes carefully selecting committee members, deciding on the values and goals of the search committee, training the committee, crafting the language of the job advertisement, and ensuring best practices for inclusive data collection and storage, among other considerations. As the search progresses, special attention should be paid to how candidates are evaluated, including through the use of standardized rubrics. Providing accessible infrastructure for candidates during visits is a must, especially on stressful interview days. For example, the stress of campus visits can be partially mitigated through practical accommodations, such as scheduling frequent breaks and providing private spaces to prepare for talks. Finally, considerations around inclusivity extend beyond selection to the offer process, and it is in the best interest of both candidates and institutions to be transparent about on-campus resources and the terms of negotiation. We provide an abbreviated list of important factors to consider in **Fig 1**, which are discussed in detail in the associated whitepaper [10].

While it is not the focus of our report, efforts to hire equitably should always be followed with systems for faculty retention. Consider how your institutions can continue supporting queer and trans candidates once we become faculty, especially in the professionally vulnerable years pre-tenure. At the same time, proactive support for queer and trans trainees at all levels is needed to ensure we make it to the stage of applying for faculty positions in the first place. Queer and trans trainees and faculty may experience significant harassment in the local community, online targeting, and even threats and physical violence. We encourage institutions to use their resources and power to protect their marginalized community members from political hostility, offering us a safe place to work. We recognize that recent legal developments in certain countries make it increasingly difficult for institutions to uphold diversity, equity, inclusion, and justice as core academic principles, but we encourage those in positions of power to take bold stances in defense of marginalized researchers rather than preemptively yielding to political pressure.

When in doubt, the best solution is always to listen to your candidates. There are many societal challenges whose roots extend beyond the scope of a faculty search committee’s responsibilities, but we nevertheless encourage you to work directly and transparently with your candidates to creatively problem solve. Working to build an equitable and inclusive search process helps everyone. Nearly all the recommendations we make can improve the experiences of all candidates, but will have an outsized effect on making queer and trans individuals feel welcome and equal in your institution.
